# Clinical Text Data Categorization and Feature Extraction Using Medical-Fissure Algorithm and Neg-Seq Algorithm

**DOI:** 10.1155/2022/5759521

**Published:** 2022-03-07

**Authors:** Naveen S Pagad, Pradeep N, Khalid K. Almuzaini, Manish Maheshwari, Durgaprasad Gangodkar, Piyush Shukla, Musah Alhassan

**Affiliations:** ^1^Department of Information Science and Engineering, Sri Dharmasthala Manjunatheshwara Institute of Technology, Ujire 574 240, India; ^2^Visvesvaraya Technological University, Belagavi, Karnataka, India; ^3^Department of Computer Science and Engineering, Bapuji Institute of Engineering and Technology, Davangere, Karnataka, India; ^4^National Center for Cybersecurity Technologies (C4C), King Abdulaziz City for Science and Technology (KACST), Riyadh 11442, Saudi Arabia; ^5^Department of Computer Science and Applications, MCNUJC, Bhopal 462003, Madhya Pradesh, India; ^6^Department: Computer Science & Engineering, Graphic Era Deemed to Be University, Dehradun, Uttarakhand, India; ^7^UIT-RGPV, Bhopal, India; ^8^University of Development Studies, Electrical Engineering Department, School of Engineering, Nyankpala Campus, Nyankpala, Ghana

## Abstract

A large amount of patient information has been gathered in Electronic Health Records (EHRs) concerning their conditions. An EHR, as an unstructured text document, serves to maintain health by identifying, treating, and curing illnesses. In this research, the technical complexities in extracting the clinical text data are removed by using machine learning and natural language processing techniques, in which an unstructured clinical text data with low data quality is recognized by Halve Progression, which uses Medical-Fissure Algorithm which provides better data quality and makes diagnosis easier by using a cross-validation approach. Moreover, to enhance the accuracy in extracting and mapping clinical text data, Clinical Data Progression uses Neg-Seq Algorithm in which the redundancy in clinical text data is removed. Finally, the extracted clinical text data is stored in the cloud with a secret key to enhance security. The proposed technique improves the data quality and provides an efficient data extraction with high accuracy of 99.6%.

## 1. Introduction

Clinical data is a standard source of information in most clinical and medical studies. Medical information is gathered either as part of routine hospital treatment or as part of a systematic clinical research plan. Clinical evidence is divided into six categories: Administrative reports, claims data, patient/disease registries, health audits, clinical trial data, and electronic health records. The purest type of electronic clinical data is collected at a treatment institution, hospital, clinic, or internship at the point of service. The electronic medical record (EMR), also known as the electronic health record (EHR), is normally not accessible to outside researchers. A longitudinal database of electronic health information about particular patients and communities is known as an electronic health record (EHR) [1]. EHRs are often used to track healthcare procedures. EHRs provide a wealth of knowledge that makes them useful for a variety of other purposes [2]. Reducing prescription mistakes, implementing improved coordination and information-sharing practices between physicians, lowering healthcare rates, better control of patients' medical records, improving care quality, and contributing to better outcomes are only a few examples.

An electronic health record is an electronic version of a patient's medical records [3] maintained by a health care professional for some time, and it includes all of the essential statistical healthcare details related to the care provided to a person by a specific provider, such as profiles, success notes, complications, prescriptions, important signs, and medical history [4]. Privacy, secrecy, and confidentiality are all concerns that must be resolved in an electronic health record system [4]. Even though security and privacy are closely linked, they are fundamentally separate. Privacy refers to a person's ability to choose when, how, and to what extent personal information is [5, 6] exchanged or transmitted by others, while confidentiality refers to the degree to which access to someone's personal information is limited and permitted. An individual's trust in the safety and confidentiality of their medical history had a positive impact on their motivation to create an electronic health record [7]. Patients' ability to encourage health care providers to exchange their medical data by using cloud computing techniques has been [8] limited as a result of privacy issues. Antivirus tools, chief information security officers, and cloud computing are other security methods that are used, but their deployment is [9] budget-dependent.

Even though the cloud storage infrastructure seems to be successful, antivirus protection remains a more widely used security measure. Security concerns have been raised as a result of IT developments such as hosting health data on remote servers managed by third-party cloud service providers [10]. Specific skills for interpreting and collecting information would be needed as information about the patient's condition continues to grow rapidly. Graphics, icons, free text, and numbers are all examples of data formats that can be contained in the EHR program [11]. There are two types of data formats: structured and unstructured. [12] Since the data already has a defined structure, traditional mathematical or machine learning approaches may be used to analyze structured data types with little effort. Hospital notes, surgical history, discharge summaries, radiology reports, diagnostic photographs, and pathology reports are the unstructured data contained in EHR.

Natural language processing (NLP) refers to a computer's capacity to comprehend the more recent human speech words and text. Natural language processing is gaining popularity in healthcare due to its ability to scan, review, and translate massive volumes of patient data. In the healthcare media, NLP will accurately give voice to the unstructured data of the universe, [13] providing incredible insight into understanding efficiency, refining processes, and improving patient outcomes. Natural language processing in healthcare employs sophisticated engines capable of scrubbing vast amounts of unstructured health data for previously ignored or incorrectly written medical conditions. Using [14–16] machine-learned algorithms to interpret medical records in natural language, an illness that could not have been coded before may be discovered. Algorithms are the building blocks in a machine learning program and are a series of instructions for completing a set of tasks. The algorithms are programmed to learn from data without the need for human interference. [17] Machine learning algorithms increase prediction accuracy over time without the need for scripting. Machine learning applications can potentially improve the accuracy of treatment protocols and health outcomes through algorithmic processes.

Thus, the analysis of unstructured data with a novel solution for data sensitivity, security, quality, and accessibility using machine learning and natural language processing should be proposed. The main goal of this research is to develop machine learning and natural language processing method for recognizing unstructured clinical text data. Even though several data extraction strategies have been proposed, recognizing the unstructured clinical text data remains difficult. The content of the paper is organized as follows: Section 1 represents the introduction; Section 2 presents the literature survey of clinical text data; the novel solutions are presented in Section 3; the implementation results and its comparison are provided in Section 4; finally, Section 5 concludes the paper.

## 2. Literature Survey

Digital Imaging and Communication in Medicine (DICOM) is considered to be the most commonly used medical image format among hospitals. Dorgham et al. [18] proposed to enhance the secure transfer and storage of medical images on the cloud by using hybrid encryption algorithms. One of today's most important priorities is the security of data processed in cloud data centers. When confidential data, such as medical images, is uploaded or shared on the cloud, it must be treated with extreme caution to ensure its reliability. They are made up of one or more compact files that cannot be seen on a screen and saved in a folder. As a result, the data can be accessed at any time. As a result, preserving data protection and denying unauthorized access becomes critical.

Agrwal et al. [19] have used a hybrid integrated Fuzzy Analytical Hierarchy Process-Technique for Order of Preference by Similarity to Ideal Solution (Fuzzy AHP-TOPSIS) method for evaluating various information security. It is essential and sufficient to evaluate information security using an integrated fuzzy MCDM methodology and to define various security attributes in a systematic and step-by-step (tree-based) fashion. This web application did not focus on data quality and data based on electronic health records.

Clinical data synthesis aims at generating realistic data for healthcare research, system implementation, and training. It is a promising tool for situations where real-world data is difficult to obtain or unnecessary. Chen et al. [20] examined an open-source well-documented synthetic data generator Synthea, which was composed of key advancements in this emerging technique. They selected a representative 1.2-million Massachusetts patient cohort generated by Synthea. Synthea and other synthetic patient generators do not use model for treatment anomalies or the possible results that could emerge from them. So synthetic data generators consider critical quality measurements in their logic and model when clinicians can deviate from the standard to produce a more practical data collection.

In recent years, deep learning techniques have demonstrated superior performance over traditional machine learning (ML) techniques for various general-domain NLP tasks. Clinical documents pose unique challenges compared to general-domain text due to the widespread use of acronyms and nonstandard clinical jargon by healthcare providers. The study by Hasan et al. [21] shows that compared to methods using linear models such as support vector machines (SVMs) or logistic regression, nonlinear neural network models have promising outcomes. The obtained state-of-the-art outcomes as opposed to the lexicon-, knowledge-source-, and conventional machine learning-based systems, demonstrating the usefulness of deep learning approaches to solve different clinical NLP issues, do not state the accessibility of unstructured data.

Identifying chronic conditions in the electronic health record is an important but challenging task. Here, systems adopt methods that allow for automated “noisy labeling” of positive and negative controls. Murray et al. [22] combined a variant of the Easy Ensemble method with the technique of Learning with Noisy Labels. Each of the individual models was trained by using all the 583 positive cases and a random pool of 583 negative patients. All the models in the ensemble were trained with 1 : 1 class balance and shared the same positive set. This is important for conditions such as systemic lupus erythematosus SLE, for which diagnostic uncertainty is common, and there is often incomplete documentation.

Kumar et al. [23] presented an overview of the current state of healthcare information and a tiered model for healthcare information management in businesses. The report also assesses the numerous elements that play a role in healthcare information security breaches. AHP-TOPSIS' hybrid fuzzy-based symmetrical technique. Furthermore, to examine the impact of the estimated results, the authors tested the results on Varanasi's local hospital software. The comparison and sensitivity analysis verify the tested outcomes of the parameters. However, the efficient and accurate extraction of clinical text data is not considered in this work.

Harnoune et al. [24] presented an end-to-end strategy for information extraction and analysis from biological, clinical notes using the Bidirectional Encoder Representations from Transformers (BERT) model and the Conditional Random Field (CRF) layer. They also constructed a named entity recognition model capable of recognizing entities such as drug, strength, duration, frequency, adverse drug responses, the rationale for taking medicine, method of administration, and form. However, the security and authority of clinical data during storage are not considered in this work.

In [18], cloud transfer of data was a tedious process [19]. Security should be maintained in clinical data [20] as sensitive information needs more privacy [21] and data quality to improve the accessibility [22] of unstructured data. [23] requires efficient and accurate data extraction and in [24], there is a need to consider the security and authority in the clinical text data. Hence, it is understood that the existing techniques face problems in improving the quality of clinical text data; the accessibility of unstructured data is not provided, and it is difficult to maintain data security and authority. Based on an overview of the literature survey, the problem faced on data security, data quality, accessibility of unstructured data should be processed, and a new novel solution had to be implemented based on machine learning and natural language processing. The proposed methods will contribute to all stages of clinical text data extraction, starting with splitting the clinical text data and ending with extraction and storage. The approaches that are already in use in clinical data extraction are explained above. The next section explains the techniques and benefits of the algorithms in the proposed method.

## 3. Discovery of Knowledge in Clinical Data Using Machine Learning and Natural Language Processing in Cloud

The machine learning approach focuses on advanced computational techniques to identify data and the natural language processing methods enabled to process and analyze textual data written in human languages. Recognition of clinical text data was a tedious process; existing techniques have used several methods for structured data but not in unstructured data, so it could not determine the effective results and data quality. Using our novel Halve Progression, we recognize unstructured clinical text data based on machine learning techniques to split the unstructured clinical text data according to the disease condition. The novel Halve Progression technique utilizes a novel Medical-Fissure algorithm that uses cross-validation based on structured data and thus, the recognition terms are made to be more efficient. After recognizing text data, extraction of data is required to obtain extensive knowledge in clinical data. This can be processed based on clinical language processing; existing techniques could not determine the ambiguity, and mapping with medical terms was not accurate. Our proposed Clinical Data Progression technique uses Neg-Seq algorithm that uses statistical features and Unified Medical Language System (UMLS) with unique identification for mapping. Hence, the resultant data can be used for further diagnosis activity. Extracted data can be stored in a cloud platform since it is considered to be best for accessibility and storage, so an effective cloud framework is required to store clinical data as it contains vast data and sensitive information. Our Cloud Progression uses RS access control that.

**Figure 1:**
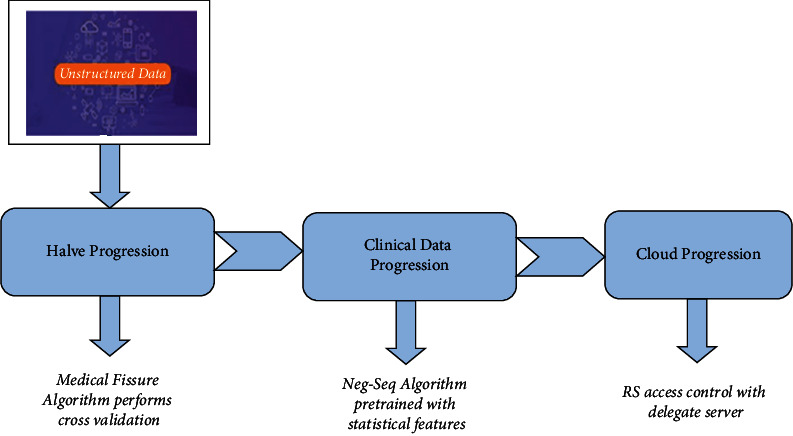
Proposed framework.

It performs validation and authorizes and has a private key for data sharing. So the clinical text data is stored in the cloud with security and authority. Hence, as shown in Figure 1, in our proposed novel method, clinical text data is recognized, extracted, and stored efficiently by machine learning techniques and natural language processing in a cloud environment.

### 3.1. Halve Progression

The clinical text data recognition was challenging, particularly unstructured clinical text data recognition, and the prior approach could not identify the effective outcome because it required many conversion procedures. A machine learning approach is employed in this work to detect unstructured clinical text data. Using a cross-validation approach, the Medical-Fissure Algorithm divides clinical text data based on illness state.

Halve progression is used to split the clinical text data towards a more advanced state, thereby increasing the data quality. Halve progression uses the Medical-Fissure algorithm for the clinical text data categorization. In the Medical-Fissure algorithm, the original clinical text data, which contains much clinical information, is divided into reduced categories of clinical text data according to some specific condition. Halve Progression based on Medical-Fissure Algorithm provides the best result with F-score in cross-validation trials, indicating the need to split the text data depending on the sick state. For example, the trained five classifiers are needed to detect arterial hypertension (AH), myocardial infarction (MI), stroke, diabetes mellitus (DM), and angina pectoris (AP). For stroke, MI, and AH, using negation classifiers is critical. The classifiers for MI and AH learn context and assist in the discovery of more examples of these illnesses. The most important words for identifying MI, including illness terminology and treatment options, are included in surgery and medications.

When the negations are recognized, a logistic loss is used to categorize each phrase in the anamnesis as containing or not containing negation. Sentences or portions of sentences with negations are deleted from anamnesis so that these texts may be utilized to create additional models that solely address the patient's current situations, such as topic modeling.

**Figure 2:**
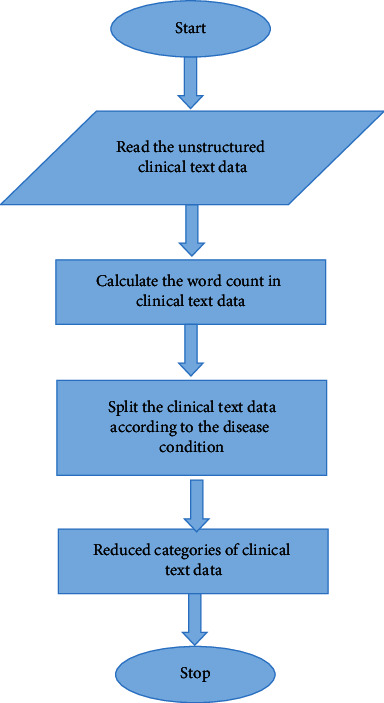
Flowchart for halve progression.

The basic goal of the Medical-Fissure Algorithm is to detect unstructured clinical text data and split it based on a unique sick state. As shown in Figure 2, First, unstructured clinical data is used as input, which implies data that does not follow any conventional format. Second, the Medical-Fissure Method calculates the count in the clinical text data, and the prerequisite for this algorithm is that the clinical text data be present in the input. Third, using a cross-validation technique, the clinical text data is separated into distinct illness conditions. Finally, the filtered clinical text data is the output of this Medical-Fissure Algorithm. As a result, data quality improves and recognition words become more efficient. After recognizing text data, data extraction is required to obtain extensive knowledge in clinical data; this can be processed using clinical language processing because ambiguity determination and mapping with medical terms were not accurate. The next subsection explains the next approach, Clinical Data Progression.

### 3.2. Clinical Data Progression

The Halve Progression improves data quality, but the mapping and extraction of medical words are ineffective. Clinical Data Progression employs the Neg-Seq Algorithm, which is pretrained using statistical characteristics, which include the size, provenance, collection methods, and annotation of the clinical text data. Statistical characteristics accurately collect data, conduct appropriate analyses, and effectively increase the efficiency of data extraction. The Neg-Seq Algorithm uses statistical features for the extraction of clinical text data. By using statistical features, the clinical text data is extracted based on medical terms. For example, if a medical term related to heart is taken means the features are extracted based on the information related to the heart, such as heart operation, heart diseases, treatments taken by the heart patients, medicines for heart diseases, etc. UMLS with unique identifiers is utilized for mapping. It is essential to eliminate any additional brackets, points, commas, colons, semicolons, dashes, hyphens, parentheses, apostrophes, quotation marks, and so on from the medical transcript. Neg-Seq Algorithm is mainly used to remove the redundant data present in the reduced categories of clinical text data obtained from Halve Progression technique. Since Neg-Seq Algorithm uses statistical features, it can detect redundant data and even redundant punctuations and thereby makes the redundant features absent in the reduced categories of clinical text data.

The Neg-Seq Algorithm is syntactically nonredundant; however, it can create semantically redundant patterns in reality. For pairings of patterns like (a b −b c) and (a −b b c), redundancy exists, and it is easy to avoid creating both effectively. To overcome this problem, the method describes the negative datasets as a collection of negative items before composing the final dataset with new items.

Mapping medical terms with Unified Medical Language System (UMLS) involves the following steps:

1. Create a class model for your development domain
2. Use the model to identify persistent classes
3. Assume that each persistent class in the model maps to one relational table
4. For each class hierarchy, choose an appropriate inheritance technique
5. Add a unique ID (OID) for each class or choose an appropriate primary key
6. Map basic data types to table columns for each class
7. Map complicated characteristics (association, aggregation) to *P*_*k*_, *F*_*K*_ pairs for each class
8. Keep an eye out for the strong and weak aggregation types
9. Map *P*_*k*_, *F*_*K*_ pairs identifying the role ends according to the specified key for associated classes
10. Classify relationship roles according to their cardinality

By using UMLS with unique identification, the major issues in mapping the clinical text data are solved and it makes the mapping more accurate.

**Figure 3:**
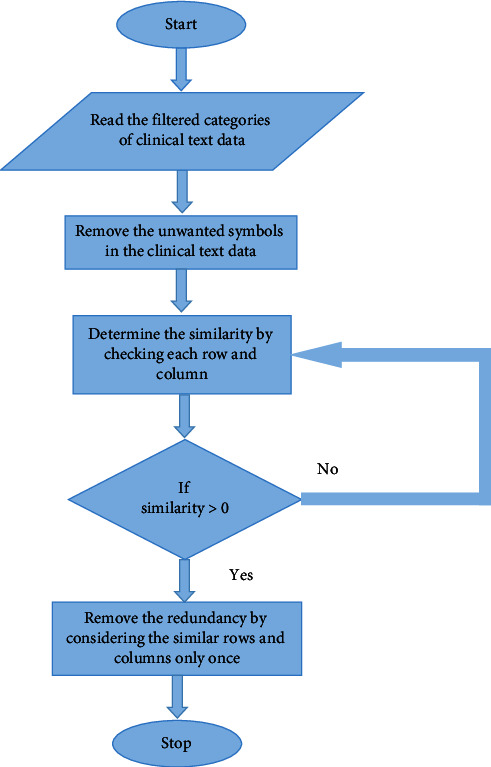
Flowchart for Neg-Seq algorithm.

The Neg-Seq Algorithm, as shown in Figure 3, improves the extraction and mapping methods by using the result of Halve Progression, which is the categorized clinical text data, as input and removing the unwanted punctuations that are repeated in the input; thus, this algorithm aims to remove redundancy in the clinical text data. The redundancy is then eliminated from every row and column. Finally, precise data is obtained. As a result, the extracted data can be used for further diagnostic purposes.

Extracted data can be stored in a cloud platform because it is the best option for accessibility and storage; however, an effective cloud framework is required to store clinical data because it contains a large amount of data and private information. The next subsection explains the next approach, Cloud Progression.

### 3.3. Cloud Progression

The data collected from the Clinical Data Progression is kept in the cloud, which should keep critical information secure. The clinical text data is saved in the cloud to improve security and authority. For storage, cloud advancement uses a framework as a service. Storage as a service refers to the practice of storing data on public cloud storage facilities. However, it needs to improve privacy; therefore, it is required to employ RS (Recommended Standard) access control, in which a delegate server performs validation and grants authorization. The delegate server acts as an intermediary and stores the security key for data exchange. The main objective of RS access control is identification, authorization, authentication, confidentiality, integrity, availability, and accountability.

**Figure 4:**
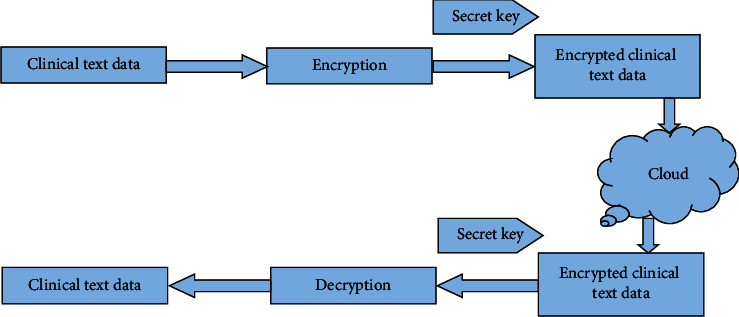
Cloud Progression using cryptography for cloud storage.

Figure 4 shows the cloud progression using cryptography for cloud storage. Cloud cryptography uses encryption techniques to protect data that will be utilized or stored there. It enables users to use shared cloud services simply and safely since all data held by cloud providers is encrypted. Cloud cryptography secures sensitive data without slowing down information flow. The encryption method encrypts data on the client-side before sending it to the cloud for storage. Plaintext will be converted to ciphertext, preventing data theft from man-in-the-middle attacks. That is, even if an attacker intercepts the data, he will be unable to read it or derive any useful information from it. This secret key is used for both encryption and decryption algorithms.

The private key is taken as <*j*, *k*> and the clinical text data is taken as ‘*t*' and the ciphertext that is the encrypted clinical text data is taken as ‘*q*'

To determine the ciphertext ‘q' the below formula is used:

(1) $$\begin{array}{c} q=t^{j}\mathrm{mod}k. \end{array}$$

To determine the clinical text data ‘*t*' the below formula is used:

(2) $$\begin{array}{c} t=q^{j}\mathrm{mod}k, \end{array}$$

where

 
*q*: encrypted clinical text data
 
*t*: original clinical text data
  <*j*, *k*>: secret key

**Figure 5:**
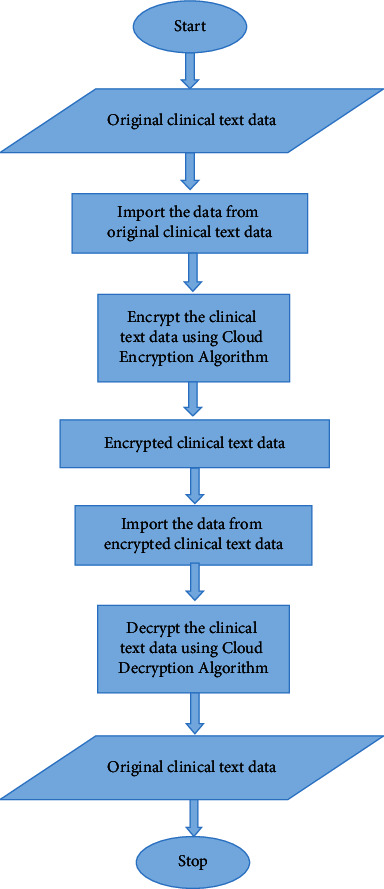
Flowchart for cloud encryption and decryption algorithm.

Decryption is the process of restoring data to its original unencrypted state after it has been rendered unreadable via encryption. Users receive encryption keys from cloud storage providers, which encrypt data. When data must be decrypted, these keys are utilized to do it safely. The hidden data is decrypted and made readable again.  Figure 5 shows a flowchart for cloud encryption and decryption algorithm.

As a result, clinical text data is securely and authoritatively kept in the cloud. This enables machine learning and natural language processing techniques to detect, retrieve, and save the clinical text data in the cloud environment efficiently. Overall, the Discovery of Knowledge in Clinical Data Using Machine Learning and Natural Language Processing includes *n* major techniques. The first is Halve Progression, which uses a Medical Fissure algorithm to split clinical data based on diseased conditions, making diagnosis easier and improving data quality. Second, Clinical Data Progression employs the Neg-Seq Algorithm, which is pretrained using statistical characteristics to extract relevant data while also increasing the significance of the mapping. Third, Cloud Progression is used to securely store data on the cloud. Thus Discovery of Knowledge in clinical data using machine learning and natural language processing provides authorization and validation to clinical text data. The next section explains the results obtain from the Discovery of knowledge in clinical data using machine learning and natural language processing in the cloud and discusses it in detail.

## 4. Results and Discussion

This segment provides a detailed description of the implementation results and the performance of the proposed system and a comparison section to ensure that the proposed system performs valuable.

### 4.1. Experimental Setup

This work has been implemented in the working platform of python with the following system specification and the simulation results are discussed below.

  Platform: Python
  OS: Windows 7
  Processor: 64 bit Intel processor
  RAM: 8 GB RAM
  Dataset: Medical Transcription (MTSamples) Dataset

#### 4.1.1. Dataset Description

The MTSamples dataset contains 5,000 sample medical transcription reports from various specialties. The dataset includes 40 medical specialties, including ‘Surgery', ‘Consult - History and Phy', and ‘Cardiovascular/Pulmonary'. Each specialization has a set of sample reports ranging from 6 to 1103 [25–27]. The medical history, diagnosis, medicines, treatment plans, vaccination dates, allergies, radiological pictures, and laboratory and test results of a patient are all kept in this dataset. By using the proposed method, these 40 categories are often divided into 21 categories (hence 1000 samples are considered for experimentation) based upon some specified conditions, that is, by splitting the clinical text data according to the disease condition using the proposed halve progression technique.

### 4.2. Results Obtained from Each Methodology

The clinical text data used as input and the obtained results from various techniques are explained in a detailed manner.

**Figure 6:**
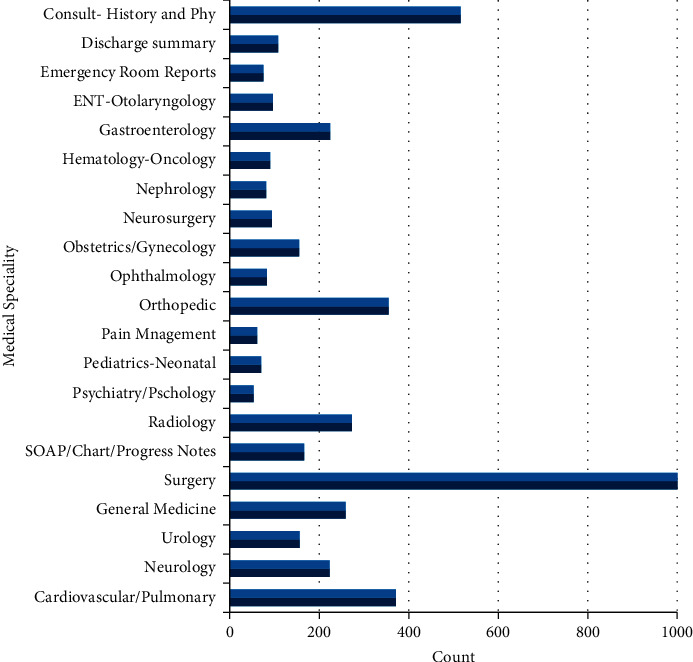
Halve progression output as reduced categories.

The MTSamplesdataset contains 40 classes that are unstructured, whereas some classes do not have any useful information for knowledge discovery from clinical data, which are not considered as a training samples. In order to exclude those uninformative classes, the halve progression technique is employed in the proposed framework. The resulted classes from 40 are 21, along with a number of records from the given 5000 record samples. The resulting 21 classes with the record count are graphically represented in Figure 6 and are statistically represented in Table 1. The records contained in those 21 classes are utilized for further processing.

### 4.3. Performance Metrics of the Proposed Method

The performance of the proposed methodology and the obtained clinical text data are detected by the following equation.

#### 4.3.1. Accuracy

The accuracy of the clinical text data is calculated using

(3) $$\begin{array}{c} \text{accuracy}=\left[\frac{\text{TP}+\text{TN}}{\text{TP}+\text{TN}+\text{FP}+\text{FN}}\right]*100. \end{array}$$

  TP: true positive value
  TN: true negative value
  FP: false positive value
  FN: false negative value

**Figure 7:**
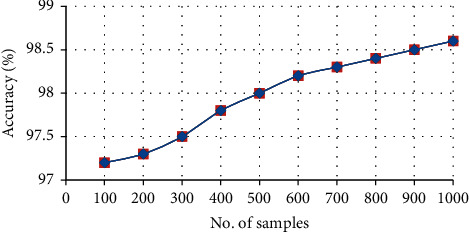
Overall accuracy of the proposed system.

Figure 7 represents the overall accuracy of the proposed system; from the graph, it is clear that the proposed system gives high accuracy with 99.6% of resultant clinical text data. The accuracy of the proposed system is increased to 99.6% by using Clinical Data Progression Approach since this approach extracts the data with statistical features, which is interpreted in Table 2.

#### 4.3.2. Specificity

Specificity is derived from the equation:

(4) $$\begin{array}{c} \text{specificity }=\frac{\text{true negative}}{\text{true negative}+\text{false positive}}\text{.} \end{array}$$

**Figure 8:**
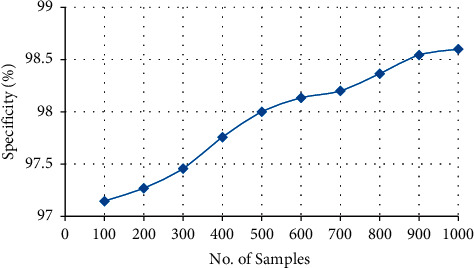
Overall specificity of the proposed system.

Table 3 and Figure 8 clearly explain the specificity of the proposed model, and the specificity of the proposed model is about 98.6%. The highest of about 98.6% specificity is attained overall by the proposed methodology. The specificity of the proposed model is increased to 98.6% by using Halve Progression approach since the quality of data is maintained by using this approach.

#### 4.3.3. Sensitivity

Sensitivity is deduced using the formula

(5) $$\begin{array}{c} \text{sensitivity}=\frac{\text{true positive}}{\text{true positive}+\text{false negative}}\text{.} \end{array}$$

**Figure 9:**
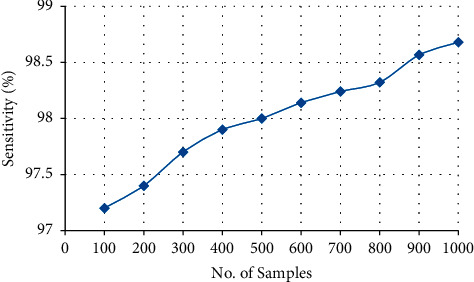
Overall sensitivity of the proposed system.

The sensitivity of the proposed is determined as 98.68%, which is illustrated in Figure 9 and Table 4. The sensitivity is overall between 97.2 and 98.68 percent. The sensitivity of the proposed system is determined by using Halve Progression approach since the recognition and division of data makes the clinical text data more sensitive.

#### 4.3.4. *F*1 Score

*F*1 Score is defined as follows:

(6) $$\begin{array}{c} \text{F}1=\frac{2\times \left(\text{precision}*\text{recall}\right)}{\text{precision}+\text{recall}}\text{,} \end{array}$$

where

(7) $$\begin{array}{c} \text{recall}=\frac{\text{TP}}{\text{TP}+\text{FN}},\\ \text{precision}=\frac{\text{TP}}{\text{TP}+\text{FP}}. \end{array}$$

**Figure 10:**
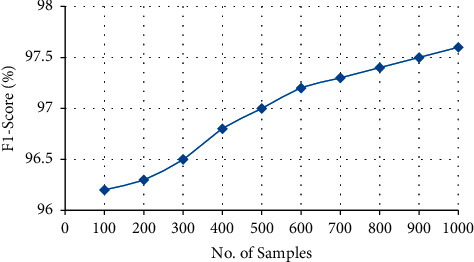
*F*1-score of the proposed system.

Table 5 and Figure 10 clearly show the *F*1 Score of the suggested model, which is about 97.6 percent. As the number of samples increases, the specificity of the model also increases. Overall, the suggested technique achieves a high level of *F*1-score of around 97.6 percent. The *F*1-score of the proposed system is determined by using the Clinical Data Progression approach in which unique identification is required.

#### 4.3.5. Precision

The closeness of two or more measurements to each other is known as precision. The formula is presented as follows:

(8) $$\begin{array}{c} \text{Precision}=\;\frac{\text{TP}}{\text{TP}+\text{FP}}, \end{array}$$

where

  TP: true positive
  FP: false positive

**Figure 11:**
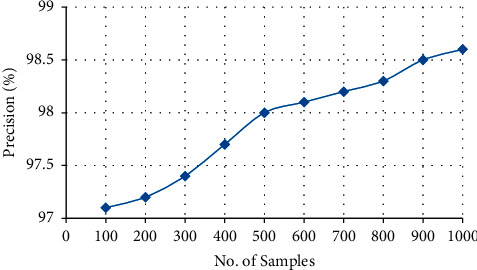
Overall precision of the proposed system.

Figure 11 represents the overall precision of the proposed system; from the graph, it is clear that the proposed system gives high precision with 98.6% of resultant clinical text data, which is listed in Table 6. The precision of the proposed method is increased to 98.6% by using the Clinical Data Progression approach since the mapping is done with the help of UMLs.

#### 4.3.6. Recall

Recall is defined as the ability of the model to accurately predict the output. The formula of recall is defined as follows:

(9) $$\begin{array}{c} \text{Recall}=\;\frac{\text{TP}}{\text{TP}+\text{FN}}, \end{array}$$

where

  TP: true positive
  FN: false negative

**Figure 12:**
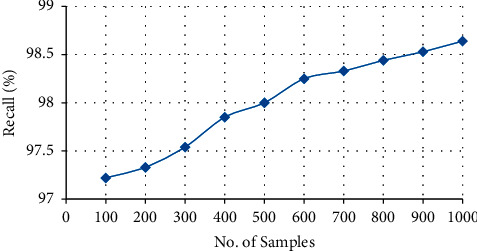
Recall of the proposed system.

From Figure 12 and Table 7, it is observed that the recalls of the proposed system are about 98.64%. Hence, the recalls increase with the increase in the number of samples. The recall of the proposed system is determined by using the Cloud Progression approach. This approach stores the entire clinical text data in the cloud with an encryption process.

This section describes the resultant performance of the proposed system. The next section describes a comparison of various performances of the previous research with the performance of the proposed method.

### 4.4. Comparison Results of the Proposed Method

This section describes various performances of the proposed method, comparing with the results of previous methodologies and depicting their results based on various metrics.

**Figure 13:**
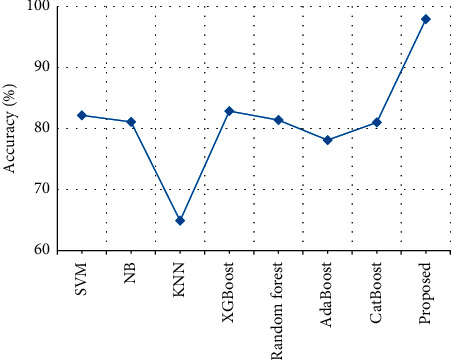
Accuracy comparison.

The accuracy of clinical text data is compared with the accuracy of the various previously proposed techniques. From Table 8 and Figure 13, it is clear that the stack accuracy of the proposed output achieves 97.9% which is 16% higher than the existing output when compared with Support Vector Machine (SVM) [27], Naïve Bayes (NB) [28], K-Nearest Neighbor (KNN) [29], XGBoost [30], Random forest [31], AdaBoost [32] and CatBoost [33].

**Figure 14:**
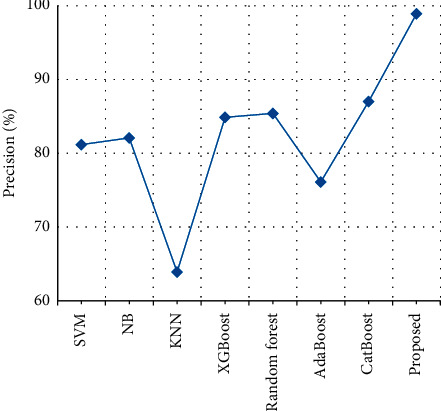
Precision comparison.

The precision of clinical text data is compared with the precision of the various previously proposed techniques. From Table 9 and Figure 14, it is clear that the stack precision of the proposed output achieves 98.9% which is 11% higher than the existing output when compared with SVM [27], NB [28], KNN [29], XGBoost [30], Random forest [31], AdaBoost [32], and CatBoost [33].

**Figure 15:**
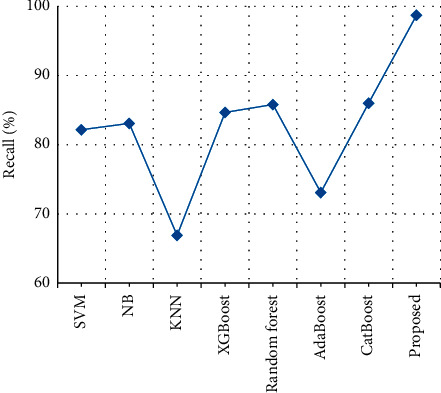
Recall comparison.

The recalls of clinical text data are compared with the recalls of the various previously proposed techniques. From Table 10 and Figure 15, it is clear that the stack recalls of the proposed output achieve 98.7%, which is 12% higher than the existing output when compared with SVM [27], NB [28], KNN [29], XGBoost [30], Random forest [31], AdaBoost [32], and CatBoost [33].

**Figure 16:**
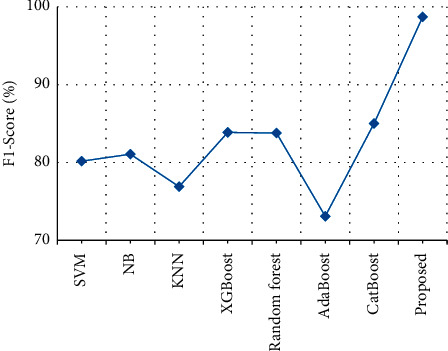
*F*1-score comparison.

The *F*1-score of clinical text data is compared with the *F*1-score of the various previously proposed techniques. From Table 11 and Figure 16, it is clear that the stack *F*1-score of the proposed output achieves 98.7%, which is 14% higher than the existing output when compared with SVM [27], NB [28], KNN [29], XGBoost [30], Random forest [31], AdaBoost [32], and CatBoost [33].

**Figure 17:**
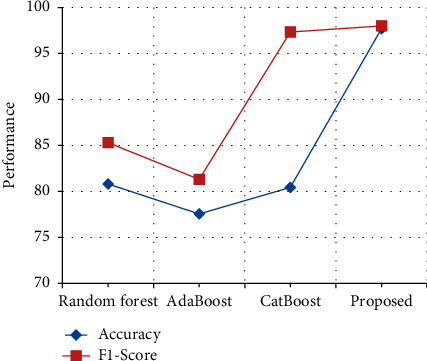
Performance in terms of accuracy and *F*1-score comparison in HoC Dataset.

The performance in terms of accuracy and *F*1-score in HoC Dataset is compared with various previously proposed techniques. From Table 12 and Figure 17, it is clear that the stack accuracy of the proposed output achieves 97.7%, which is 17% higher than the existing output when compared with Random forest [31], AdaBoost [32], and CatBoost [33], and the *F*1-score of the proposed output achieves 98% which is 1% higher than the existing output when compared with Random forest [31], AdaBoost [32], and CatBoost [33].

**Figure 18:**
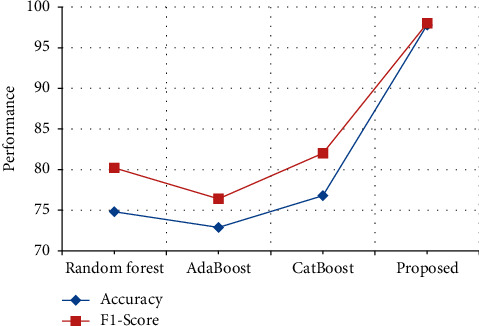
Performance in terms of accuracy and F1-score comparison in ChemProt dataset.

The performance in terms of accuracy and *F*1-score in the ChemProt Dataset is compared with various previously proposed techniques. From Table 13 and Figure 18, it is clear that the stack accuracy of the proposed output achieves 97.8%, which is 19% higher than the existing output when compared with Random forest [31], AdaBoost [32], and CatBoost [33] and the *F*1-score of the proposed output achieves 98% which is 16% higher than the existing output when compared with Random forest [31], AdaBoost [32], and CatBoost [33].

**Figure 19:**
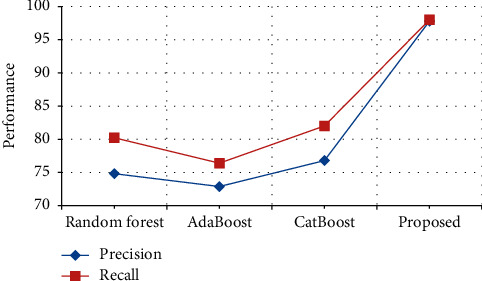
Performance in terms of precision and recall comparison in ChemProt Dataset.

The performance in terms of precision and recall in the ChemProt dataset is compared with various previously proposed techniques. From Table 14 and Figure 19, it is clear that the precision of the proposed output achieves 97.85%, which is 22% higher than the existing output when compared with Random forest [31], AdaBoost [32] and CatBoost [33], and the recall of the proposed output achieves 98.8% which is 17% higher than the existing output when compared with Random forest [31], AdaBoost [32] and CatBoost [33].

**Figure 20:**
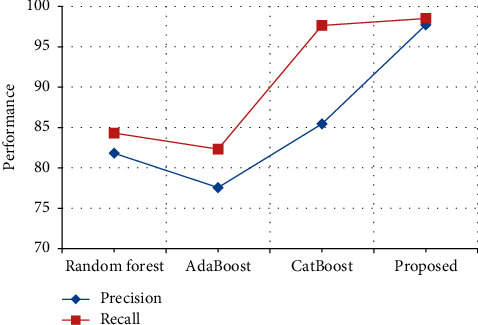
Performance in terms of precision and recall comparison in HoC Dataset.

The performance in terms of precision and recall in the HoC Dataset is compared with various previously proposed techniques. From Table 15 and Figure 20, it is clear that the precision of the proposed output achieves 97.71%, which is 12% higher than the existing output when compared with Random forest [31], AdaBoost [32] and CatBoost [33], and the recall of the proposed output achieves 98.5%, which is 1% higher than the existing output when compared with Random forest [31], AdaBoost [32], and CatBoost [33]. Thus, the proposed method functions are proved to have the best performance by comparing with results of previous research.

## 5. Conclusion

The technical complexities in extracting the clinical text data are removed by using machine learning and natural language processing techniques. Halve Progression, Clinical Data Progression, and Cloud Progression provide a solution for major issues like difficulty in diagnosis, reduced data quality, difficulty in extraction and mapping, and risk in security by using Medical-Fissure Algorithm to split the clinical text data and Neg-Seq Algorithm to remove redundancy and usage of the secret key to provide better security. The clinical text data is extracted with high accuracy of 99.6%. The results of the proposed method are compared with other existing techniques and the proposed method outperforms all the other existing techniques. To further improve the quality and accuracy of data extraction, the relation extraction using Transformer based models in clinical text data can be developed for knowledge discovery.

## Figures and Tables

**Algorithm 1:**
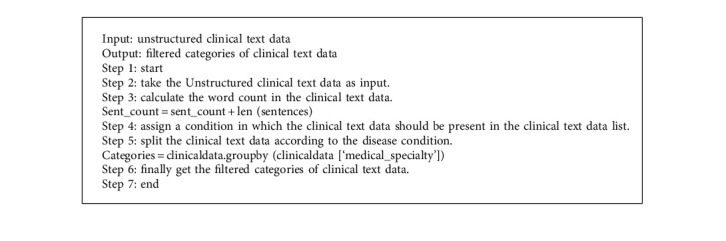
Medical-Fissure Algorithm.

**Algorithm 2:**
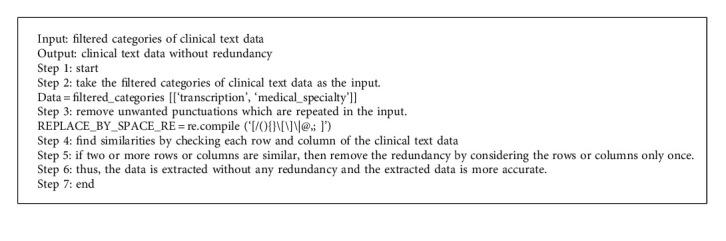
Neg-Seq Algorithm.

**Algorithm 3:**
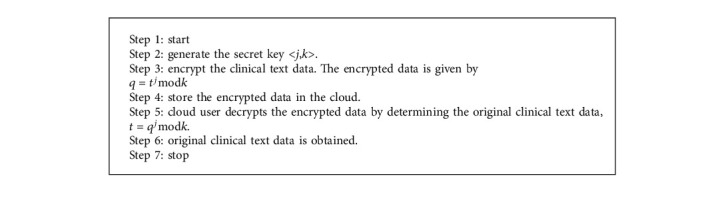
Cloud Encryption Algorithm and Decryption Algorithm.

**Table 1:** Statistical representation of halve progression output as reduced categories.

Medical speciality	Statistical count
Cardiovascular/Pulmonary	371
Neurology	223
Urology	156
General medicine	259
Surgery	1088
SOAP/Chart/Progress notes	166
Radiology	273
Psychiatry/Pschology	53
Pediatrics-neonatal	70
Pain management	61
Orthopedic	355
Ophthalmology	83
Obstetrics/Gynecology	155
Neurosurgery	94
Nephrology	81
Hematology-oncology	90
Gastroenterology	224
ENT-otolaryngology	96
Emergency room reports	75
Discharge summary	108
Consult- history and phy	516

**Table 2:** Accuracy of the proposed system.

No. of samples	Accuracy
100	97.2
200	97.3
300	97.5
400	97.8
500	98
600	98.2
700	98.3
800	98.4
900	98.5
1000	98.6

**Table 3:** Specificity of the proposed system.

No. of samples	Specificity
100	97.146
200	97.27
300	97.457
400	97.757
500	98
600	98.134
700	98.2
800	98.365
900	98.544
1000	98.6

**Table 4:** Sensitivity of the proposed system.

No. of samples	Sensitivity
100	97.2
200	97.4
300	97.7
400	97.9
500	98
600	98.14
700	98.24
800	98.322
900	98.566
1000	98.68

**Table 5:** *F*1-score of the proposed system.

No. of samples	*F*1-score
100	96.2
200	96.3
300	96.5
400	96.8
500	97
600	97.2
700	97.3
800	97.4
900	97.5
1000	97.6

**Table 6:** Precision of the proposed system.

No. of samples	Precision
100	97.1
200	97.2
300	97.4
400	97.7
500	98
600	98.1
700	98.2
800	98.3
900	98.5
1000	98.6

**Table 7:** Recall of proposed system.

No. of samples	Recall
100	97.22
200	97.33
300	97.54
400	97.85
500	98
600	98.25
700	98.33
800	98.44
900	98.53
1000	98.64

**Table 8:** Accuracy comparison.

Methodologies	Accuracy
SVM	82.17
NB	81.08
KNN	64.9
XGBoost	82.87
Random forest	81.4
AdaBoost	78.1
CatBoost	81
Proposed	97.9

**Table 9:** Precision comparison.

Methodologies	Precision
SVM	81.17
NB	82.08
KNN	63.9
XGBoost	84.87
Random forest	85.4
AdaBoost	76.1
CatBoost	87
Proposed	98.9

**Table 10:** Recall comparison.

Methodologies	Recall
SVM	82.17
NB	83.08
KNN	66.9
XGBoost	84.67
Random forest	85.8
AdaBoost	73.1
CatBoost	86
Proposed	98.7

**Table 11:** *F*1-score comparison.

Methodologies	*F*1-score
SVM	80.17
NB	81.08
KNN	76.9
XGBoost	83.87
Random forest	83.8
AdaBoost	73.1
CatBoost	85
Proposed	98.7

**Table 12:** Accuracy and F1-score comparison in HoC Dataset.

HoC dataset	Methodologies	Accuracy	*F*1-score
	Random forest	80.82	85.31
	AdaBoost	77.56	81.32
	CatBoost	80.45	97.34
	Proposed	97.7	98

**Table 13:** Accuracy and *F*1-score comparison in ChemProt Dataset.

ChemProt dataset	Methodologies	Accuracy	*F*1-score
	Random forest	74.82	80.22
	AdaBoost	72.88	76.40
	CatBoost	76.78	82.01
	Proposed	97.8	98

**Table 14:** Precision and recall comparison in ChemProt Dataset.

ChemProt dataset	Methodologies	Precision	Recall
	Random forest	74.87	80.72
	AdaBoost	72.48	76.10
	CatBoost	76.68	81.01
	Proposed	97.85	98.8

**Table 15:** Precision and Recall comparison in HoC Dataset.

HoC dataset	Methodologies	Precision	Recall
	Random forest	81.82	84.31
	AdaBoost	77.56	82.32
	CatBoost	85.45	97.64
	Proposed	97.71	98.5

## Data Availability

The data that support the findings of this study are available upon request from the corresponding author.
